# Decreased cellular excitability of pyramidal tract neurons in primary motor cortex leads to paradoxically increased network activity in simulated parkinsonian motor cortex

**DOI:** 10.21203/rs.3.rs-6254909/v1

**Published:** 2025-04-11

**Authors:** Donald W Doherty, Liqiang Chen, Yoland Smith, Thomas Wichmann, Hong-yuan Chu, William W Lytton

**Affiliations:** SUNY Downstate Medical Center; Georgetown University Medical Center; Emory University; Emory University; Georgetown University Medical Center; SUNY Downstate Medical Center

## Abstract

Recent evidence suggests that the primary motor cortex (M1) layer 5B pyramidal tract (PT5B) neurons show a decreased intrinsic excitability in mouse models of parkinsonism, which perhaps plays an important role in the pathophysiology of parkinsonian motor symptoms. PT5B neurons project to outputs in the brainstem and the spinal cord, leading to the direct motor expression of Parkinson’s disease (PD) pathology. We set out to explore how the decreased PT5B neuron excitability influences the activity patterns of the M1 network. Using NEURON/NetPyNE simulators, we implemented detailed computer simulations of PT5B neurons based on control and 6-OHDA-treated mouse slice data. We placed these PT5B cells in an *in vivo* M1 network simulation, driven by ascending input from the thalamus and from other cortical areas. Simulated 6-OHDA-treated mouse PT5B neurons in an otherwise unmodified simulated M1 network resulted in major changes in LFP oscillatory power in the parkinsonian condition: an order of magnitude increase in beta band power around 15 Hz in the rest state and a lesser increase in beta power in the parkinsonian activated (movement) state. We demonstrated that relatively small changes in PT5B neuron excitability altered the patterns of activity throughout the M1 circuit. In particular, the decreased PT5B neuron excitability resulted in increased beta band power, which is a signature of PD pathophysiology.

## Introduction

Neurodegeneration in the substantia nigra pars compacta (SNc) is the best known neuropathology in Parkinson’s disease (PD)^1^. It results in reduced dopamine (DA) levels in the brain and is closely linked to the motor disability of the disease^2^. However, as with any brain diseases, causes are multifactorial. Any circuit perturbation leads to a series of compensatory mechanisms at the site of the initial damage, and in brain areas that are directly or indirectly connected to it. In the case of SNc degeneration, these areas include the basal ganglia, thalamus, sensorimotor cortex, and cerebellum. In the classic model of PD pathology, the loss of DA due to SNc degeneration resulted in basal ganglia inhibition of activity in the motor thalamus which resulted in decreased activity in primary motor cortex (M1) and, therefore, in hypokinetic motor symptoms.^2^

Because the cerebral cortex provides a final common pathway of motor commands from telencephalon to brainstem and spinal cord, pathology in cortex, whether early or late, is likely to play a disproportionate role in the pathophysiology of parkinsonism, particularly with respect to disabilities of fine-motor tasks.^3^ In a recent study, Chu and colleagues showed a decrease in excitability of pyramidal tract neurons in layer 5B (PT5B) of M1 in a DA-depleted mouse model of parkinsonism.^4^ We hypothesized that this decreased PT5B neuron excitability would substantially disrupt oscillatory and non-oscillatory firing patterns of neurons in layer 5 (L5) of M1. To test this hypothesis, we performed computer simulations using a previously validated computer model of the mouse M1^5^ that incorporates 15 neuron types, each with detailed multicompartment, active-dendrite Hodgkin-Huxley-style structure. We studied both a resting and activated state, with the activated state being simulated as an increase M1 thalamic input and through the effects of norepinephrine on dendritic hyperpolarization-activated cyclic nucleotide–gated (HCN) channels.^5,6^

We found that inclusion of the experimentally identified parkinsonism-associated decrease of PT5B excitability into our computational model produced, paradoxically, an increase of the PT5B firing in the resting state. There was also a change in firing pattern, expressed as a major increase in beta-band oscillatory beta-band oscillatory power in PT5B neurons in the parkinsonian condition compared with the control model. During activation there was no parkinsonism-associated change in the PT5B population firing rate but changes in their firing patterns were expressed through the appearance of 20–35 Hz rhythmic activity (corresponding to the beta and low gamma range) in the parkinsonian, but not the healthy, model. The appearance of beta-band oscillations in the parkinsonian condition may be sufficient to disrupt normal M1 motor output and contribute to motor activity deficits seen in patients with PD.

## Methods

We utilized our previously developed network simulations of the mouse M1^5^, written using the NEURON/NetPyNE simulation platform,^7–9^ to compare the control “healthy” condition with a model of the parkinsonian condition based on the reduction of the PT5B cells excitability demonstrated in the 6-hydroxydopamine treated, dopamine-depleted mice.^4^ A 64% decrease in excitability (similar to the experimental findings; supplementary Fig. 1 or Fig S1) was simulated using an increase in the density of K^+^ (BK) and Na+ (NaT) channels (Table S2). We ran 4 complete sets of experiments (Table S1), each set including the control and parkinsonian conditions in the rest state and the control and parkinsonian conditions in the activated (movement) state. Each set used unique random seeds for connectivity, stimulation, and neuron location. In this paper we used the experiment set sM1_12-12-2023_01 as a typical example. Analyses of the 3 other sets of experiments are shown in Supplemental Data Figs S3-S13.

**Figure 1:**
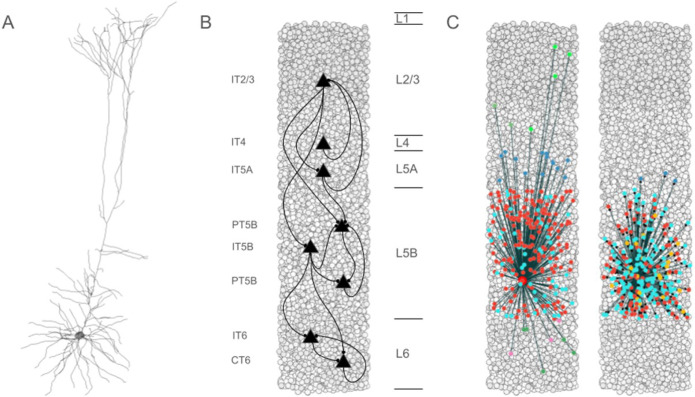
Simulated primary motor cortex (M1), composed of >10,000 neurons. A. Simulated layer 5 pyramidal tract neuron (PT5B) morphology, using >700 compartments with multiple ion channel mechanisms; B. Pyramidal cell connectivity across layers; C. Convergence and divergence example from single deep PT5B neuron (other PT5B: red, layer 2/3 somatostatin inhibitory neuron (SOM2/3): light green, layer 2/3 parvalbumin inhibitory neuron (PV2/3): dark green, layer 5A PV (PV5A): dark blue, layer 5B SOM (SOM5B): orange, layer 5B PV (PV5B): light blue, layer 6 SOM (SOM6): pink, layer 6 PV (PV6): dark green).

Seven excitatory pyramidal cell types and two interneuron cell types were simulated in the network (Fig. 1). Our detailed multicompartment model for PT5B was based on prior layer 5B PT *in vitro* electrophysiological studies of the responses of these cells to somatic current injections.^10^ The models included various subtypes of excitatory pyramidal cells such as PT, intratelencephalic (IT) and corticothalamic (CT) neurons (Fig. 1B), while inhibitory model neurons included parvalbumin- (PV-) and somatostatin- (SOM-) containing interneurons. In the following text and figures, the abbreviated names are followed by the corresponding layer number including 2/3 (layers 2,3 together) and 5A vs 5B. Although M1 is classified as agranular cortex, we included layer 4 cells, based on previous experimental studies.^11^

In the M1 simulation, neuronal activities were driven by ascending input from ventromedial thalamus (VM) to layer 2/3 (L2/3), L4, L5A neurons and also from the ventrolateral thalamus (VL) onto L4, L5B; from primary and secondary somatosensory cortices (S1 and S2) to L2/3, L5A; from contralateral primary (cM1) and ipsilateral secondary motor cortices (M2) to L5B, L6; and from orbital cortex (OC) to L6 as described in the original model^5^. Each input region consisted of a population of 1000 spike-generators (NEURON VecStims) that generated independent random Poisson spike trains (based on experimental background activity: VL 0–2.5 Hz; VM of 0–5 Hz; S1, S2, OC 0–5 Hz; cM1, M2 0–2.5 Hz).

The M1 model in the current study was identical to M1 validated in the Dura-Bernal et al. (2023) paper except in the current study control PT currents were slightly modified (Nap, NaT, and BK; Table S2) so that their response properties were more similar to the mean control PT neurons reported by Chen et al. (2021). In the simulated parkinsonian mouse M1 PT currents (BK and NaT) were modified so that they showed a 64% decrease in excitability as reported by Chen et al. (2021).

The resting (quiet wakefulness) and activated (movement) states were simulated as validated by Dura-Burnal et al. (2023). In particular, the activated state was simulated by increasing thalamic inputs from motor thalamus to 0–10 Hz (uniform distribution), and reducing Ih conductance to 25% in PT5B neurons, to simulate a high level of norepinephrine input from the locus coeruleus (LC). The other inputs continued to provide unchanged drive.

We used unitary event analysis for identifying synchronous spiking significantly above the expected number of synchronous spikes for the neuron population size and firing rates.^12^

We used the local field potential (LFP) method built into the NetPyNE framework. LFP was calculated at each simulated electrode using the line source approximation^13,14^, which is based on the sum of the membrane current source generated at each cell segment divided by the distance between the segment and the electrode. The calculation assumes that the electric conductivity (sigma = 0.3 mS/mm) and permittivity of the extracellular medium are constant everywhere and do not depend on frequency. We collected LFP signals from extracellular electrodes located at multiple depths within the M1 simulation.

Duty cycle is a common concept in electrical engineering and electronics and is defined as the ratio of time a load or circuit is on compared to the time the load or circuit is off. The use of the term has a rich history in neurophysiology^15–17^ and in motor system neurophysiology in particular^18–21^ where duty cycle often refers to the proportion of time the neuron or neural circuit is actively firing within a given period but can refer to other on-off cycles such as contracted versus relaxed muscle tissue. Duty cycle has also been defined as on when a threshold is achieved and otherwise off if not^17^.

In the current study, duty cycle is defined as on when 50% activity is reached from the trough (minimum activity) to the peak (maximum activity) during one period as measured in a spike histogram. A period is defined as the duration from the time increasing activity crosses the 50% activity threshold to the time that decreasing activity crosses the same threshold.

## Results

Biophysically-realistic, empirically-validated simulations utilized a mouse M1 network model composed of more than 10,000 neurons comprising PT, IT, PV, SOM neurons that were distributed across 6 cortical layers in a 300 μm diameter cylindrical volume (Fig. 1).^5,10^ Recent comparison of model results with in vivo mouse M1 recordings and behavior validated the data driven M1 model’s cell-type specific responses during resting (quiet) and activated (movement) behaviors.^5^ In addition, local field potential (LFP) results were validated through the analyses of their sources.^5^ In the present study, over 100,000 simulations were run in developing and exploring these simulations; one second of simulation time took about 2 hours to compute on a 5.16 peak petaflops 64-node supercomputer. The simulations were done under resting and activated states, as defined above.^5^

### Control simulations

**Figure 2:**
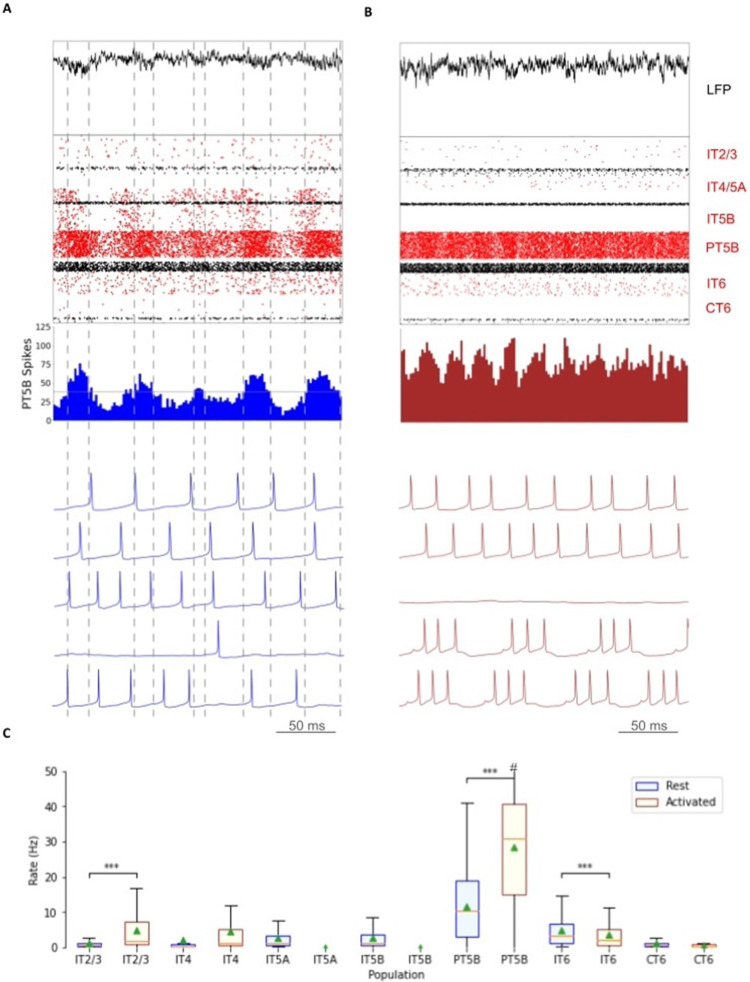
Control condition. A. and B. show the resting state on the left, and the activated state on the right. A. Top-to-bottom: local field potential (LFP), raster plot (red excitatory; black inhibitory neurons); PT5B spike count histograms (left: dashed lines at half height). B. Examples of PT5B voltage traces -- same 5 (out of 1435) in both states. C. Firing rate boxplot (mean, std dev, range, *p<0.05, **p<0.01, ***p<0.001). #, maximum firing rate in activated state: 73.0 spikes/s.

**Figure 3:**
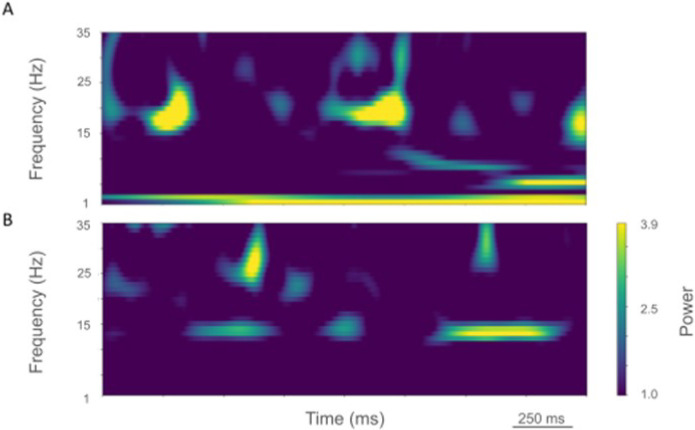
Time-resolved spectrograms of cortical LFPs in the control condition, under rest (A) and activation (B) states. Power in bars × 1.0e-5.

Comparison of rest and activated states in the control condition revealed dominant beta-band activity (~ 20 Hz) in the rest state that transitioned to gamma-band activity (~ 44 Hz) with the motor thalamic input changed to activated-state activity (Fig. 2). In the rest state, each layer 5 pyramidal cell population (IT5A, IT5B, PT5B) showed different rates and durations of spiking activity with respect to each of the other populations during beta-band oscillations (Fig. 2A). IT5A neurons were active from around the low point of PT5B activity, visible in the blue PT5B spike count histogram (SCH) at bottom of Fig. 2A, and increased to around peak PT5B activity. IT5B neurons were primarily active during the increase in PT5B activity, from around the beginning of the PT5B activity duty cycle (see below), to around peak PT5B activity (Fig. 2A, PT5B-SCH). In contrast, in the activated state, thalamic drive onto IT4 neurons lead to activation of PT5B and a decrease to zero activity in IT5A and IT5B (Fig. 2B). The activated state showed greater gamma oscillation, seen in the LFP signal (Fig. 2AB, top; also see Fig. 3B) as high frequency low-amplitude periodic deflections with intermittent periods of oscillatory activity at higher amplitude (~ 44 Hz; Fig. 2B). The beta frequency band in the LFP signal during rest and the gamma-band activity during the activated state were reflected in antiphase oscillations in their respective PT5B-SCH.

PT5B neurons greatly increased their spiking overall, and changed their firing pattern in the activated state (Fig. 2C). We analyzed the coefficient of variation (CV) of interspike intervals (ISI) observed across the PT5B neuron population to identify patterns of firing. The CV was found to be highly variable across the PT5B population during rest (0.59 +/− 0.37), indicating the presence of both regular (CV < = 0.5; 47.8%) and irregular activity (CV > 0.5; 52.2%). Using the same definitions, PT5B populations showed primarily irregular spiking in the activated state, again with high variability between neurons (overall CV 0.61 +/− 0.30; 25.7% CV < = 0.5; 74.3% CV > 0.5). Oscillations in subthreshold membrane potentials appeared frequently during the activated state (57.4% of PT5B neurons) but not during rest (Fig. 2AB). Prominent excitatory postsynaptic potentials (EPSPs) appeared before the first spike in oscillatory traces in the activated state but not in the rest state or in non-oscillatory traces in the activated state.

We explored oscillatory activity patterns further, identifying the oscillation period as beginning during increasing spike counts where the spike number was halfway between the minimum and maximum spike counts (Fig. 2AB; horizontal gray line in PT5B-SCH gives half height; dashed vertical lines show half-height at rising and falling; distance along x-axis between 2 vertical lines is a single period). Each period was divided into high activity (above half-height) versus low activity (below) sections. We defined a duty cycle as the percentage of time during one oscillation period with relatively high activity (above half-height). The duty cycle was 31% during rest (Fig. 2A) and 48% during activated state (Fig. 2B; dashed vertical lines not shown).

Pyramidal tract neurons had the highest spike rate of any neuron population during the rest state, and increased further in the activated state (rest: 11.7 ± 9.4 spikes/s; activated: 26.8 ± 18.2 spikes/s; p < 0.001;Fig. 2C). In contrast, IT5A and IT5B firing rates decreased from 3 Hz (IT5A: 3.3 spikes/s; IT5B: 3.1 spikes/s) at rest, to 0 spikes/s in the activated state, and IT6 firing rates decreased from 5.8 spikes/s at rest to 3.5 spikes/s in the activated state (p < 0.001). IT2/3 neurons significantly increased their firing rate (rest: 1.2 spikes/s; activated: 6.6 spikes/s; p < 0.001). The IT4 neuron population firing rate increased from 2.6 Hz during rest to 4.7 Hz during the activated state.

The rest state simulation revealed virtually continuous power around 2 Hz, as well as 15–25 Hz beta-band bursts (Fig. 3A), occurring every 600 ms (or with a burst-rate of 1.7 Hz). During the activated state the 2 Hz activity disappeared and beta bursts shifted to 15 Hz bursts ~ 100–500 ms in duration or about 1.5 Hz. Beta-band power between 15–20 Hz was no longer visible (Fig. 3B). Higher frequency brief bursts of activity (~ 50 ms duration) 25–35 Hz occurred once every 1000 ms or about 1 Hz.

**Figure 4:**
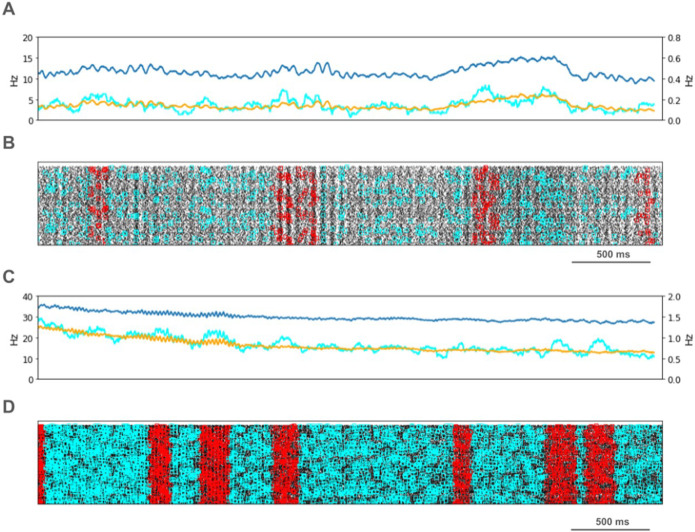
PT5B Control condition: A. Resting state: coincidence rates (cyan; left y-axis) exceed expected (orange) which tracks overall rate (blue; right y-axis). B. Resting state: coincident events (cyan) and periods of significant coincident events (* p < 0.05; joint-surprise) with individual events in red^22^. C. Activated: coincidence rates (cyan; left y-axis) exceed expected (orange) which tracks overall rate (blue; right y-axis). D. Activated: coincident events (cyan) and periods of significant coincident events (* p < 0.05; joint-surprise^22^; 100ms firing rate window^40^) with individual events in red.

We looked for spikes that fired in the PT5B neuron population within a 1 ms time window of one another and considered those to be coincident spikes. The proportion of spikes that were coincident with that of other PT5B neurons within 1 ms was substantially higher in the activated M1, as compared to simulation of M1 at rest (Fig. 4). PT5B spike coincident firing was observed in 1% of spikes at rest and increased to 3% during the activated state (Fig. 4, cyan lines in A,C cyan + red in raster in B,D). PT5B coincident firing that exceeded those expected from frequency-matched random Poisson processes (significance at p < 0.05; joint-surprise test^22^) was seen during rest in 100–275 ms duration clusters (red squares in Fig. 4B). The mean periodic activity during significant coincident events (red bands) was 19 Hz as measured during each period (red band) of significant synchrony. The range of periodic activity across individual bands of significant synchrony was 15–24 Hz. During the activated state, more frequent and denser clusters or periods of synchrony were seen than during the rest state, with durations of 125–250 ms (red squares in Fig. 4D). The total duration of significant synchronous activity increased from 0.7 s out of 4.0 s (17.5%) at rest to 1.1 s out of 4.0 s (27.5%).

### Parkinsonian condition

**Figure 5:**
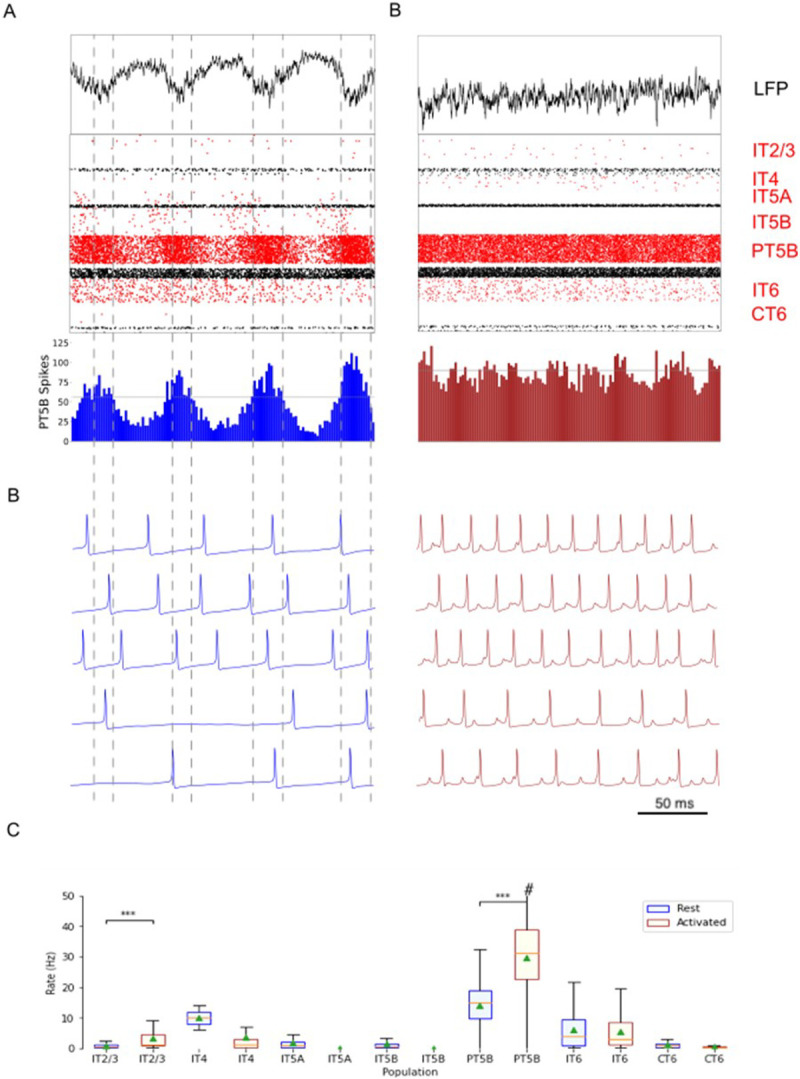
Parkinsonian condition. A. and B. show the resting state on the left, and the activated state on the right. A. Top-to-bottom: LFP record, raster plot (red pyramidal; black inhibitory neurons), spike count histograms (PT5B; 2 ms bins). B. Examples of PT5B voltage traces. C. Firing rate boxplot (mean, std dev, range, *p<0.05, **p<0.01, ***p<0.001). #, maximum firing rate in activated state: 65.7 spikes/s.

**Figure 6:**
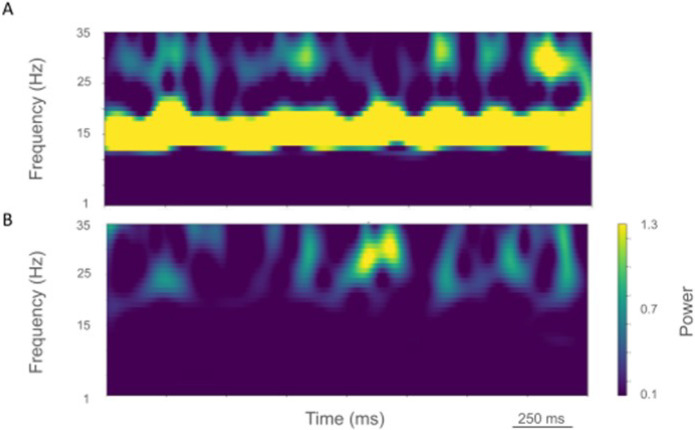
Time-resolved spectrograms of LFPs in M1 in the parkinsonian condition. A. resting state, B. activated state. Color coding shown in colorbar (x 1.0e-4).

Comparison of the simulated rest and activated states in the parkinsonian condition revealed the presence of focused 15 Hz beta-band activity in the rest state that transitioned to gamma-band activity (~ 43 Hz) in the activated state (Fig. 5). In the rest state, the layer 5 pyramidal cell populations (IT5A, IT5B, PT5B) showed beta band oscillations with slightly different phases (Fig. 5A, raster diagrams): IT5A and IT5B neurons were active during the leading phases in the PT5B-SCH oscillation (Fig. 5A). In contrast, in the activated state, the thalamic drive onto IT4 neurons led to activation of PT5B neurons, and stopped activity in IT5A and IT5B. The activated state showed greater gamma oscillation in the simulated LFP signal (Fig. 6A), as higher frequency low-amplitude periodic deflections with intermittent higher amplitude excursions (~ 44 Hz; Fig. 6B). The beta frequency band in the LFP signal during rest and the gamma-band activity during the activated state were reflected in antiphase oscillations in their respective PT5B-SCH.

PT5B neurons both greatly increased spiking and changed firing pattern in the activated state (Fig. 5C). The CV of the PT5B neuron population ISIs analysis showed a peak signifying regular activity (CV of 0.34 +/− 0.27) but with a large enough standard deviation to include irregular activity in the PT5B population during rest: regular (80.6% CV < = 0.5) and irregular activity (19.4% CV > 0.5). In the activated state, PT5B population analysis showed regular spiking that included a small number of irregular spiking neurons (CV 0.18 +/− 0.19; 93.8% CV < = 0.5 regular; 6.2% CV > 0.5 irregular). Subthreshold EPSPs were clearly evident during the activated state but not during resting (Fig. 5AB). No subthreshold membrane potential oscillations were observed in the parkinsonian rest or active states. The duty cycle was 29% during the parkinsonian rest state (Fig. 5A) and 25% during activated state (Fig. 5B; dashed vertical lines not shown).

Pyramidal tract neurons had the highest spike rate of any neuronal population during the rest and activated states in the parkinsonian condition (rest: 14.2 ± 7.1 spikes/s; activated: 29.7 ± 12.7 spikes/s; p < 0.001; Fig. 5C). IT5A and IT5B firing rates decreased from 2.2 and 1.2 spikes/s, respectively, at rest to 0 spikes/s in the activated state. The firing rates of IT6 neurons did not change (rest and activated: 6.0 spikes/s). In contrast, superficial layer IT2/3 neurons significantly increased their average firing rates with activation (rest: 0.8 spikes/s; activated: 4.3 spikes/s; p < 0.001).

LFP spectrograms of resting oscillatory power displayed continuous high-power 15 Hz beta-band power with bursts of power in the ~ 25–35 Hz (~ 50–250 ms duration) occurring about once every 200 ms (~ 5.0 Hz; Fig. 6A). In the activated state, ~ 20–35 Hz bursts ~ 25–100 ms in duration were seen that occurred every~ 50–100 ms (~ 10–20 Hz; Fig. 6B).

**Figure 7:**
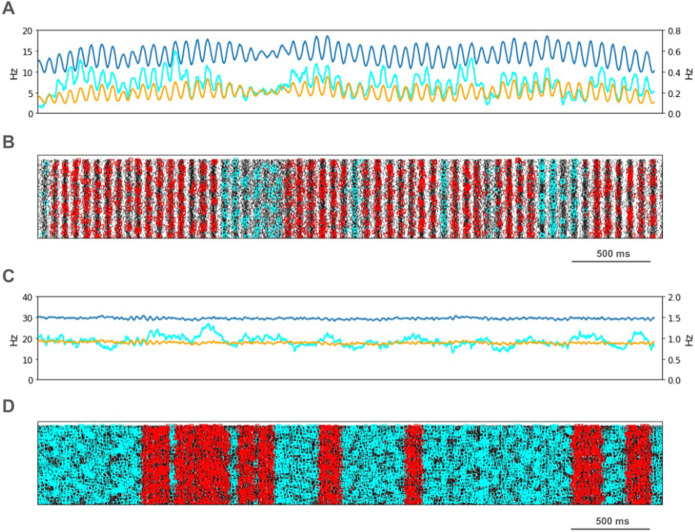
PT5B Parkinsonian condition: A. Resting state: coincidence rates (cyan; left y-axis) exceed expected (orange) which tracks overall rate (blue; right y-axis). B. Resting state: coincident events (cyan) and periods of significant coincident events (* p < 0.05; joint-surprise) with individual events in red. C. Activated: coincidence rates (cyan; left y-axis) exceed expected (orange) which tracks overall rate (blue; right y-axis). D. Activated: coincident events (cyan) and periods of significant coincident events (* p < 0.05; joint-surprise^22^; 100ms firing rate window^40^) with individual events in red.

PT5B spike coincident firing (2 or more spikes firing in 1ms) was observed in 2% of spikes at rest and increased to 3% during the activated state (Fig. 7, cyan lines in A,C cyan + red in raster in B,D). PT5B coincident firing that exceeded those expected from frequency-matched random Poisson processes (p < 0.05; joint-surprise test^22^) was seen during rest in 400–1,100 ms duration clusters with a periodic structure within each, consisting of 6 to 16 periods (red squares in Fig. 7B). The mean periodic activity during significant coincident events (red bands) was 15 Hz as measured during each period (red band) of significant synchrony. The range of periodic activity across individual bands of significant synchrony was 14–15 Hz. During the activated state, the significant PT5B coincident firing (p < 0.05) occurred in epochs of dense synchronous activity, 100–350 ms in duration (red squares in Fig. 6B). The total duration of significant synchronous activity during rest was 3.1 s out of 4.0 s (77.5%). During the activated state, 1.4 s of 4.0 s (35.0%), was significantly synchronous.

### Control vs parkinsonian comparison

The most substantial changes in firing between conditions were seen in the simulated rest state, where there was a significant increase in PT5B firing rate and a decrease in firing rate variability in the parkinsonian condition as compared with control condition (control: 11.7 ± 9.4 spikes/s; parkinsonian: 14.2 ± 7.1 spikes/s; p < 0.001). There was also an increase in IT4 firing rate (control: 2.6 spikes/s; parkinsonian: 14.1 spikes/s; p < 0.05) and a decrease in IT5A firing rate (control: 3.3 spikes/s; parkinsonian: 2.2 spikes/s; p < 0.01).

Activated (movement-associated) cortical dynamics also showed a significant increase in PT5B firing rate and a decrease in firing rate variability in the parkinsonian condition as compared with control condition (control: 26.8 ± 18.2 spikes/s; parkinsonian: 29.7 ± 12.7 spikes/s; p < 0.001). The firing rate of IT6 neurons also increased in the parkinsonian condition (control: 3.5 spikes/s; parkinsonian: 6.0 spikes/s; p < 0.001).

## Discussion

We performed a series of simulations based on one of several cortical changes that have been found to take place in parksionian animal models in rodents,^4,23^ and non-human primates,^24,25^ comparable to what is seen in patients^26,27^ (recently reviewed in^27^). We examined the effects of reduced PT5B intrinsic cellular excitability in parkinsonian mice. In the resting state, reduced PT5B neuron excitability resulted in a paradoxical increase in PT5B firing in the network condition, as well as an increase in beta oscillatory power with reduced frequency, increased PT5B spike synchrony, and firing rate shifts in other cell populations. Parkinsonism-associated changes were less marked in the activated state; we found no significant change in PT5B population firing rate but a change in PT5B activity pattern as expressed by an increase in power of 20–35 Hz activity.

### Rest versus Activated

Shifts in dominant frequency activity were prominent in resting versus activated data. At rest we observed 2 Hz oscillations and 15–20 Hz beta-band bursts in LFP signals once every ~ 0.6 s. In contrast, during the activated state, only the lowest frequency beta bursts (~ 15 Hz) in the 15–20 Hz range remained and high beta and low gamma oscillation bursts appeared in the 25–35 Hz band. These findings are in line with previous results indicating that beta-rhythms are known to desynchronize during voluntary movement.^28,29^ Within this context, it is interesting that our simulation of M1 neuronal activity during the activated state consisted of only two changes: 1) an increase in random spikes from thalamus from 5 Hz to 10 Hz, and 2) a decrease in H current in PT5B neurons. These relatively small changes were sufficient to result in decreased power across most beta band rhythm frequencies in M1.

### Effects of the parkinsonian condition

The parkinsonian condition was marked by the appearance of vigorous 25–35 Hz band bursts in both the resting and activation states. At the resting state, an order of magnitude increase in beta power was observed around 15 Hz. With activation, the high-beta and low-gamma oscillation bursts (25–35 Hz) extended to lower frequencies around 20 Hz. As mentioned, these changes were produced by a decrease of the excitability of cortical neurons, simulated using an increase in the density of K^+^ channels.

### Role of beta oscillatory activity in parkinsonism

In healthy individuals, an early hypothesis was that beta-oscillatory bursts in M1 (as identified by EEG or electrocorticogram recordings) were thought to be a marker of an idling state before movement is initiated.^28,29^ Brief desynchronization of beta oscillatory activity is associated with voluntary movement, only to return once movement is completed.^28,30^ A more recent hypothesis is that beta band activity has a stabilizing effect, signifying active processes that promote existing motor set while suppressing the neuronal processing of new movements.^29,31^ which is compatible with the status quo hypothesis. The frequency of beta oscillations is strongly coupled with the dopamine tone in monkeys and humans^30,32^ Lesions of midbrain dopaminergic neurons in animals lead to an increase in beta-frequency oscillatory activity in the basal ganglia, M1^24^, subthalamic nucleus, and globus pallidus.

Many studies have reported an increase in beta band power or in beta-oscillatory bursts in the basal ganglia, thalamus, or cortex of patients with PD or in animal models of dopamine loss^24,25,32^. A connection between DA loss and beta activity was demonstrated in rodents when deep brain stimulation in parkinsonian rats destroyed the M1 dominance of beta rhythms and restored motor control^33^. In addition, a recent study demonstrated beta oscillations in the basal ganglia of parkin knockout mice^34^.

The overall amount of beta-band power in LFP signals is now considered to reflect the average of beta burst^35^ activity during the period that was examined. Although not universally agreed upon,^36^ there may be several key differences between the beta burst activity in healthy and parkinsonian subjects. Thus, the timing and duration of beta power bursts is highly variable in healthy subjects, while the variability is much lower in patients with severe PD. Further, in contrast to findings in healthy subjects, beta bursts in M1 in parkinsonian individuals are often unusually long (even greater than 100 ms duration^35^). It has been suggested that the brevity of beta bursts in the healthy state could be critical to normal beta-band function.^35^

Waveform features of beta oscillations in LFP (or electrocorticogram) signals may reflect synchronous excitatory synaptic inputs onto cortical pyramidal neurons. Beta-band oscillations in M1 in parkinsonian patients have sharp, asymmetric, nonsinusoidal features that are correlated with beta-high gamma phase-amplitude coupling. The observation of sharp beta oscillations in PD M1 due to synchrony of synaptic activity has been hypothesized to be due to increases in beta synchrony in the basal ganglia. We observed changes in synchrony and significant increases in beta oscillations in simulated M1 with only small changes in potassium and sodium currents and only in PT5B neurons. Observed changes were intracortical changes in activity patterns due to M1 circuitry and biophysics.

### Synchronous activity in parkinsonism

Oscillations in the brain provide an effective means to control the timing of neuronal firing. Cortical neurons support highly precise and reliable spike times to naturalistic fluctuating inputs^37^ and are good detectors of correlated activity.^38^ Oscillations can temporally coordinate information transfer and support spike-timing dependent plasticity.

Significant increases in synchronous neural activity in M1 is consistently observed in PD^39^ or in animal models of the parkinsonian condition, including an increase in the concurrence of beta bursts^36^. We found a substantial increase in synchronous spiking in our simulations of the parkinsonian rest state.

### Study limitations

Major limitations of this study are the limitations that are inherent in all modeling studies—we necessarily made choices as to what to include and what to leave out. Many parameters are not considered since they have not been studied experimentally or cannot currently be studied in detail (this includes, e.g., most functions of dendritic spines). In particular, (1) we did not consider interneuron populations other than PV and SOM cells; (2) we did not consider the cortical effects of dopamine; (3) we modeled inhibitory neurons as single compartments; (4) we have incomplete models for the distribution of voltage- and calcium-sensitive dendritic channels in pyramidal dendrites. We qualitatively matched PT5B data in control and 6-OHDA conditions by manually modifying conductances. Finally, we modeled a model of a model: the *in vitro* slice, which itself is a model of the 6-OHDA *in vivo* rodent model of human parkinsonism.

## Conclusions

Relatively small local changes in cortical excitability suffice to induce changes in cortical physiology that resemble the parkinsonian condition. This change occurred as a consequence of a clear-cut local intervention (6-OHDA treatment), that most likely has most of its effects on basal ganglia activity rather than cortex directly. The current study demonstrates that the effects of the subcortical dopamine loss may not be the ‘transmission’ of abnormal subcortical signals to the cerebral cortex, but that M1 DA-depletion leads to a decrease in PT5B excitability which is a sufficient change in the cortical circuit to result in generating abnormal M1 oscillatory activities which may, in turn, alter basal ganglia activity patterns.

## Data Availability

The software used to generate data and carry out this study is openly available in GitHub at http://doi.org/10.5281/zenodo.12399983.
